# Supplementary material for: Association of Mediterranean Diet Scores with Psychological Distress in Pregnancy: The Japan Environment and Children’s Study
Source: Nutrients. 2025 Nov 25;17(23):3697. doi: 10.3390/nu17233697 (PMC12693845; doi:10.3390/nu17233697)
Supplement: Supplementary file 1 [file nutrients-17-03697-s001.zip › Table S1_2025.11.pdf]

Table S1. Maternal alcohol intake during pregnancy.

Alcohol intake (ethanol equivalent), g/day	N (%)
**0**	79,013 (98.4%)
**0.1–4.9**	982 (1.2%)
**5–25 (1point for MDS, rMED)**	244 (0.3%)
**>25**	32 (0.04%)

MDS: Mediterranean Diet Score and rMED: relative Mediterranean Diet.
