# Supplementary material for: Association of Mediterranean Diet Scores with Psychological Distress in Pregnancy: The Japan Environment and Children’s Study
Source: Nutrients. 2025 Nov 25;17(23):3697. doi: 10.3390/nu17233697 (PMC12693845; doi:10.3390/nu17233697)
Supplement: Supplementary file 1 [file nutrients-17-03697-s001.zip › Table S2_2025.11.pdf]

Table S2. Proportion of each MDS and rMED score. (a) Proportion of each MDS score; (b) Proportion of each rMED score.

MDS score	N (%)
**0**	5 (0.01)
**1**	220 (0.27)
**2**	3,379 (4.21)
**3**	21,415 (26.68)
**4**	35,784 (44.58)
**5**	17,948 (22.36)
**6**	1,321 (1.65)
**7**	176 (0.22)
**8**	23 (0.03)
**9**	0 (0.00)

(a)

rMED score	N (%)
**0**	1 (0.00)
**1**	12 (0.01)
**2**	157 (0.20)
**3**	791 (0.99)
**4**	4,205 (5.24)
**5**	7,397 (9.22)
**6**	13,010 (16.21)
**7**	14,450 (18.00)
**8**	14,386 (17.92)
**9**	11,511 (14.34)
**10**	7,866 (9.80)
**11**	4,209 (5.24)
**12**	1,715 (2.14)
**13**	475 (0.59)
**14**	85 (0.11)
**15**	1 (0.00)
**16–18**	0 (0.00)

(b)

MDS: Mediterranean Diet Score and rMED: relative Mediterranean Diet.
